# 112. Prescriber Perceptions on Utilization of the Antibiotic Self-Stewardship Time Out Program (SSTOP) at Veterans Affairs Medical Centers (VAMC): A Strategy for Improved Antibiotic Use

**DOI:** 10.1093/ofid/ofab466.314

**Published:** 2021-12-04

**Authors:** Cassie Goedken, Joshua Judd, Jorie M Butler, Nui G Brown, Michael Rubin, Matthew B Goetz, Matthew B Goetz

**Affiliations:** 1 VA Iowa City Health Care System, Iowa City, IA; 2 VA Salt Lake City Health Care System, Salt Lake City, Utah; 3 University of Utah, Salt Lake City, UT; 4 VA Greater LA Healthcare System, Greater LA, California; 5 IDEAS Center of Innovation, VA Salt Lake City Health Care System, Salt Lake City, Utah; 6 VA Greater Los Angeles Healthcare System and David Geffen School of Medicine at UCLA, VA-CDC Practice-Based Research Network, Los Angeles, California

## Abstract

**Background:**

Evidence is lacking on how to implement effective and sustainable antibiotic stewardship strategies. The Antibiotic Self-Stewardship Time Out Program (SSTOP) evaluated the implementation at VAMCs of an “Antibiotic Timeout” 3 days after the initiation of antibiotics to encourage providers to review continued use of broad-spectrum antibiotics.

**Methods:**

Sites launched the SSTOP note templates in a rolling fashion from June 2019-March 2020. Clinical pharmacists largely drove the implementation. The vancomycin note template was implemented at 6 of 8 sites and the antipseudomonal note template across 4 of 8 sites. Two sites were unable to launch the note templates due to lack of resources, however they utilized SSTOP principles/guided tools. From Sept 2019-Nov 2020 we conducted post-launch qualitative interviews with Antibiotic Stewardship Program (ASP) champions involved in implementation across the 8 VAMCs. Interviews were transcribed and analyzed for thematic content.

**Results:**

Feedback from ASP providers suggests prescribers had mixed reviews on the note template, but overall liked the process and deemed it to be straightforward. Many valued the algorithm, indicating it was helpful in both thinking about antibiotics prior to initiation, and identification of appropriate antibiotics. Barriers included staffing (e.g., rotating residents/turnover), surgery service, information technology (IT) support, COVID-19, and the need to remind providers to use the template. Facilitators consisted of strong stewardship, local champions (e.g., ID Fellow), medicine service, and SSTOP data feedback reports. Recommendations largely centered on improvements to the note template usability and to SSTOP feedback reports (e.g., inclusion of patient/provider-level data).

**Conclusion:**

Overall, the SSTOP note templates were considered acceptable and straightforward. By guiding providers to prescribe more appropriate antibiotics, they act as influencers for practice change, and may strengthen provider/ASP relations. Plans for continued utilization of the note templates after the project concludes suggest SSTOP may serve as a way to achieve sustainable promotion of antibiotic use improvements.

**Disclosures:**

**Matthew B. Goetz, MD**, Nothing to disclose

